# A comment on the paper ‘A comparison between lesions found during meat inspection of finishing pigs raised under organic/free-range conditions and conventional indoor conditions’ by Alban et al. 2015

**DOI:** 10.1186/s40813-016-0031-4

**Published:** 2016-06-09

**Authors:** Jan Tind Sørensen

**Affiliations:** Department of Animal Science, Aarhus University, Blichers Alle 20, DK-8830 Tjele, Denmark

**Keywords:** Pig health, Production system, Meat inspection

## Abstract

This is a critical comment on a paper published in Porcine Health Management in 2015, presenting a comparison between lesions from meat inspection at one abattoir on slaughter pigs classified in to two different production forms: organic/free-range conditions and conventional indoor conditions. The conclusion made by the paper that 13 lesion types has a higher prevalence in organic/free-range pigs and 4 lesion types occurred less frequently in organic/free-range finishers compared to conventional finishers is correct except that 5 (instead of 4) lesion types occurred less frequently in organic/free-range finishers. However, these five types of lesions represent 74 % of all lesions recorded among conventional indoor, conventional free-range and organic pigs in one slaughter house from October 1 2012 to September 26 2013.

## Background

Organic pork offers an alternative to the consumer because the production form is different from indoor conventional production with better opportunities to express normal behaviour and using a low level of antibiotics [1]. The consumer, however, expect that animal welfare in general is better in organic pig production than in conventional indoor pig production [2]. A Danish study on pig morbidity based on meat inspection data showed that organic pigs had a higher level milky spots in liver indicating a higher level of endoparasite infections but a lower level of chronic pleuritis compared to indoor pigs [3]. A recent paper [4] had been widely commented in Danish media’s concluding that organic pigs are more diseased than indoor pigs. The conclusion made in this paper [4] can, however, be discussed.

## Discussion

The paper [4] presents a comparison between lesions from meat inspection at one abattoir on slaughter pigs classified in to two different production forms: organic/free-range conditions and conventional indoor conditions.

A Chi-square comparison is made using the meat inspection protocol for all lesion types. Further, an inclusion criterion for presenting the results is that the prevalence should be at least 0.1 % for one of the two production forms. In total results is presented for 21 lesion types. The biological importance of the differences between the two systems is considered if the odds ratio (OR) is above 1.2 “higher risk” or below 0.8 “lower risk” and the *P* value is less than *P* = 0.0001. If OR is between 0.8 and 1.2 then the level is classified as “equal risk”.

The authors present the differences in production requirements between the two, rather different, systems; conventional free-range pig production and organic pig production. It appears from the requirements that stocking density is twice as high in conventional free-range compared to organic and that the requirements focusing on antibiotics is different. In fact the use of antibiotics is quite different between organic pigs and conventional free-range pigs, whereas the use of antibiotics for conventional free-range pigs and conventional indoor pigs is almost similar [5]. Due to these differences between conventional free-range pig production and organic pig production these two systems should have been considered separately. In the study period 1/10-2012 -26/09-2013 the number of organic pigs slaughtered were 97,600 and the number of conventional free-range pigs were 103,600 (Friland A/S personally communication 2015). The authors state the number of organic and conventional free-range farms included in the study, but they do not state that less than half of the organic /free-range pigs were organic [4].

The conclusion stated in the paper [4] is:

- ’Compared with conventional pigs organic/free-range pigs has a higher prevalence on 13 lesion types’

And further:

‘Finally four lesions occurred less frequently in organic/free-range finishers compared to conventional finishers’.

From Table 2 it appears that the odds ratio for lesions is 1.34 in organic/free-range pigs compared to conventional pigs (when chronic pleuritis is excluded).

The use of an odds ratio as only indication on whether the differences reflect a biological association could be discussed. Study limitations make that results from Alban et al. should be examined cautiously, and that causation should not be deduced from the statistical associations observed. Further it is questionable to treat chronic pleuritis similar to all other lesion types because chronic pleuritis account for more than half of all lesions included in the study and all other lesion types had prevalence of lesions below 4 %.

It is stated in the analysis that odds ratio for chronic pleuritis is between 0.8 and 1.2 and therefore is classified as ‘equal’. It appears from Table 2 that:

- Number of chronic pleuritis in organic/free-range pigs is: 38,344 (79,174 – 40,830)
- Number of chronic pleuritis in conventional indoor pigs is: 280,811 (459,239 – 178,428)

Total number of pigs included in the study is:

- Organic/free-range:210,160
- Conventional indoor:1,173,213

Based on this information odds ratio for chronic pleuritis in an organic/free-range pig compared to a conventional indoor pig can be calculated. The exact odds ratio is 0.748, which is less than 0.8.

According to the authors own classification of “higher”, “equal”, “lower” risk of a lesion type in organic/free-range compared to conventional chronic pleuritis should have been classified as “lower” risk.

If we look at the prevalence of all lesions listed in Table 3 (all 21 lesions across the two groups of pigs (organic/free-range and conventional indoor)), and calculate total number of lesions in Table 3 divided by total number of pigs included in the study the prevalence is 38.60 %. The total prevalence of the lesions across all pigs, which is classified as lesion types with “lower” risk in organic /free-range pigs (including chronic pleuritis), is 28.65 %. Thus, organic/free-range pigs had a “lower” risk to be detected with 5 types of lesions that represents 28.65/38.60 = 74 % of all individual lesions recorded.

This is a conclusion, which differs from the conclusion made by the authors.

## Conclusion

Organic/free-range pigs had a lower risk to be detected with 5 types of lesions (including chronic pleuritis) that represented 74 % of all individual lesions recorded among conventional indoor and organic/free-range pigs in one Danish slaughterhouse from October 1, 2012 to September 26, 2013.
